# Supplementary material for: Variant-specific introduction and dispersal dynamics of SARS-CoV-2 in New York City – from Alpha to Omicron
Source: PLoS Pathog. 2023 Apr 18;19(4):e1011348. doi: 10.1371/journal.ppat.1011348 (PMC10180688; doi:10.1371/journal.ppat.1011348)
Supplement: Attachment — Submitted filename: Response_to_Reviewers.pdf [file ppat.1011348.s001.pdf]

Editors

Two reviewers have seen the paper, both agree there is merit here and that the method and analyses are worthy of publication. Yet both raise some concerned both by possible biases in sequencing data, and by tree topology uncertainty. Notably there is also some reliance on NextStrain sampling - how would this impact the results. I would be happy to see a revised version that addresses all the below comments.

Answer: We sincerely thank the Reviewers for their positive assessment of our work and all their constructive comments on the previous version of our manuscript, as well as the *PLoS Pathogens* editorial team for having given us the opportunity to submit a revised version of our study. We have now revised our study in response to the Reviewers' and Editor comments (see below for a point-by-point response to all comments) and hope it can now be found suitable for publication in *PLoS Pathogens*. In particular, we have performed the following main changes:

- As pointed out by the first Reviewer, there was an issue with the convergence of our Thorney BEAST analyses to time-calibrate our maximum likelihood phylogenetic trees. In addition to a mistake with the files that were shared on the GitHub repository of the study, it appeared after investigation that the subsequent discrete and continuous phylogeographic analyses were indeed based on Thorney BEAST analyses that had not optimally converged. We have tried to re-run longer Thorney BEAST analyses to reach adequate MCMC convergence and mixing for all four variants, but these attempts were not successful. To remedy this issue, we have built on our previously published analytical pipeline (Dellicour *et al*. 2021, PMID: 33316043) to redo the time-scaled phylogenetic inferences as well as subsequent discrete and continuous phylogeographic analyses. Specifically, we now use the method implemented in the program TreeTime (Sagulenko *et al*. 2018, PMID: 29340210) for the time-calibration step.
- As detailed below in our answer to the related comment of the second Reviewer, considering more than one time-scaled phylogeny is computationally compromised given the size of our datasets (involving >60,000 sequences gathered in the alignment for the Delta variant). Switching to the methodology implemented in TreeTime for the time-calibration step has now allowed us to rely on previous work to discuss the limitation of basing the phylogeographic reconstruction on a unique time-scaled phylogeny. Indeed, the robustness of phylogeographic estimates to the selection of the maximum likelihood starting tree had been assessed in Dellicour *et al*. (2021, PMID: 33316043; see below), and the results of this assessment confirmed that the different estimates remain robust irrespective of the choice of the starting tree.
- Regarding the use of previous Nextstrain builds to obtain a background set of genomic sequences that are representative of the phylogenetic diversity of the SARS-CoV-2 lineages at the corresponding point in time for each variant, we now explicitly justify that choice in our Materials and Methods section: *"The purpose of the inclusion of such a background data set is to allow placing NYC clades in a broader global context of SARS-CoV-2 phylogenetic diversity. While we cannot exclude that the selection and/or size of the background data set could influence the subsequent delimitation of NYC clades introduced into the study area, the same approach has here been applied to each variant under consideration, which guarantees a form of consistency in the clade delimitation: if an underestimation of the delineated clades applies, it should similarly affect all four variants and still allow further comparisons of their introduction dynamics."*
- As suggested by the first Reviewer, we have now implemented, conducted and reported the results of simulations performed to assess the sensitivity of our new metrics to various introduction and dispersal scenarios (see below).

Reviewers

Part I - Summary
Please use this section to discuss strengths/weaknesses of study, novelty/significance, general execution and scholarship.

Reviewer #1: In this study the authors propose a set of metrics to compare the introduction and dispersal dynamics of the main VOI/VOCs that spread across the NYC area. The metrics are based on a phylogeographic approach, including both discrete and continuous phylogeographic implementations. They apply these metrics to large sets of SARS-CoV-2 genomes and find that interesting differences in the introduction and dispersal dynamics of Iota, Alpha, Delta and Omicron.

Reviewer #2: This generally well written and well performed study by Delicour and colleagues develops and implements a novel comparative framework for phylogeographic analysis of SARS-CoV-2 variants of concern. The authors apply their framework to understand the dynamics of different variants of concern in the New York City area. What makes this paper a novel significant contribution is the analytical framework. This framework is likely to be of great interest to others. A weakness of the study is the dependence on single ML topologies for each variant. Inherent in SARS-CoV-2 phylogenies is substantial phylogenetic uncertainty, it would be prudent to condition analyses on a modest distribution of trees. The figures are useful and appropriate display items to illustrate the main results and caveats are acknowledged especially those with regard to the focal NYC dataset.

Answer: We agree with the Reviewer that performing a fully integrated phylogenetic-phylogeographic inference — or at least phylogeographic analyses based on a representative set of trees sampled from the posterior distribution of a time-scaled phylogenetic inference — would be the ideal procedure as this would enable assessing the impact of phylogenetic uncertainty on the phylogeographic analyses. Yet, performing such integrated analyses or obtaining a representative set of time-scaled posterior trees from an initial Bayesian phylogenetic inference is computationally infeasible given the size of our datasets (>60,000 sequences gathered in the alignment for the Delta variant). As an illustration of the computational burden associated with data sets of that size, the maximum-likelihood phylogenetic inference performed in IQTREE for the Delta and Omicron (BA.1) variants have respectively required more than 130 and 195 hours of running time on a dedicated high-performance computing workstation. We have now explicitly acknowledged and justified this aspect in our Discussion section:

"As detailed in previous work (Dellicour et al. 2021), we also acknowledge that an analysis where the phylogenetic tree and ancestral locations are jointly inferred would be preferable to explicitly take into account the uncertainty associated with Bayesian phylogenetic inference. When dealing with alignments gathering tens of thousands of genomic sequences, as is the case in the present study (up to more than 60,000 sequences in the Delta alignment), such fully integrated Bayesian analyses are not feasible in a reasonable amount of time. As an illustration of the computational burden associated with data sets of that size, the maximum-likelihood phylogenetic inference performed in IQTREE for the Delta and Omicron (BA.1) variants have respectively required more than 130 and 195 hours of running time on a high-performance computing workstation (40-core/64GB RAM Dell Precision workstation). In that context and while it comes with the limitation of not assessing the effect of phylogenetic uncertainty on the outcomes, using an empirical time-scaled phylogenetic tree represents an interesting compromise to run phylogeographic analyses on large-scale data sets. In our study based on much smaller data set of genomic sequences, we and other colleagues assessed the robustness of their results (inferred number of introduction events into the study area, estimates of lineage dispersal velocity) to the selection of the maximum-likelihood starting tree, and confirmed that the different estimates remain robust to the choice of the starting tree (Dellicour et al. 2021)."

Part II – Major Issues: Key Experiments Required for Acceptance
Please use this section to detail the key new experiments or modifications of existing experiments that should be absolutely required to validate study conclusions.

Generally, there should be no more than 3 such required experiments or major modifications for a "Major Revision" recommendation. If more than 3 experiments are necessary to validate the study conclusions, then you are encouraged to recommend "Reject".

Reviewer #1: The introduced metric seem intuitive and the reported results are quite believable, however, the authors are introducing their new metrics p1, p2 and p3 here without having tested them. The performance of these metrics under a small range of simulated scenarios needs to be evaluated. For that it should be sufficient to simulate a small set of introduction and dispersal scenarios, from which sequence data can be generated which is then analysed in the same fashion as presented here. This could also be used to (i) test how much data is required to robustly infer the introduction and dispersal dynamics and (ii) test how sensitive the metrics are to biased sampling.

Answer: We thank the Reviewer for this important comment and relevant suggestion. We have now implemented, conducted and reported the results of simulations we performed to assess the sensitivity of our new metrics to various introduction and dispersal scenarios:

"Besides the weighted diffusion coefficient that was previously introduced and applied (Trovão et al. 2015b), the present study thus describes three other metrics p_1-p_2-p_3 to characterise and compare the introduction/dispersal dynamics of (viral) lineages in a study area. To assess the sensitivity of those metrics to different introduction and dispersal scenarios, we simulated the invasion of the Alpha variant while testing (i) different values for the minimum size of the introduced clusters circulating in the study area (Fig. 4A-B), (ii) different numbers of introduced clusters (Fig. 4C-D), and (iii) different total numbers of transition events between counties for that variant (Fig. 4E; ***see the Material & Methods section for further detail on the simulation processes****)."*

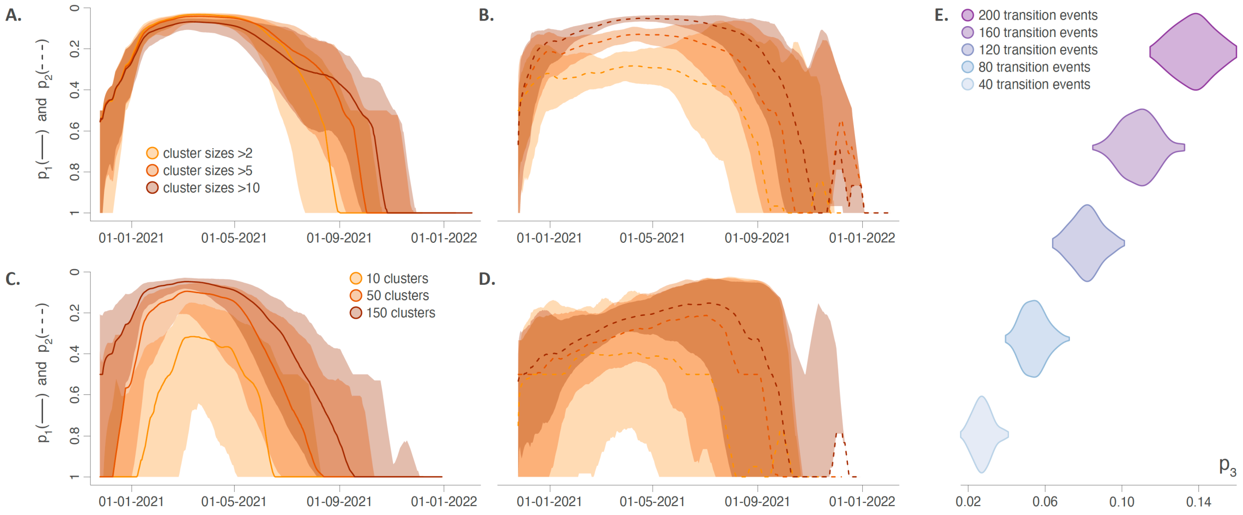

Figure 4. Sensitivity of the new metrics to different introduction and dispersal scenarios simulated for the Alpha variant. A-B: *Impact of the size of the clusters introduced into the study area on the metrics p_1 and p_2.* ***C-D:*** *Impact of the number of clusters introduced into the study area on the metrics p_1 and p_2. In panels A-D, mean p_1-p_2 values and associated 95% HPD intervals are reported by solid/dashed curves and surrounding shaded polygons, respectively.* ***E:*** *Impact of the total number of transition events (among counties) on the p_3 metric.*

"The minimum size of the introduced clusters had a negligible impact on the p_1 metric (Fig. 4A): around the peak of the epidemic wave, the probability p_1 that two circulating lineages drawn at random belong to the same cluster tends to zero in all cases. On the contrary, varying the same simulation parameter had a notable impact on the p_2 metric (Fig. 4B): the ratio p_2 between the co-occurring numbers of clusters and phylogenetic branches clearly tends to increase with the minimum introduced cluster size considered in the simulations. The number of simulated clusters introduced in the study area influenced both p_1 (Fig. 4C) and p_2 (Fig. 4D) metrics. In particular, drastically decreasing the number of introduced clusters provokes a shift of the p_1 curve, making it closer to the pattern of the p_1 curve

reported for the Iota variant (Fig. 3A), i.e. the variant that was thus associated with a major clade circulating in the study area. Finally, as expected, our results confirm that the p_3 metric is very sensitive to the number of simulated transition events among counties (Fig. 4E).”

The code written to conduct and analyse those simulations has been added to the overall R script “shared on the GitHub repo of our study: https://github.com/sdellicour/new_york_variants. This overall R script now includes two new sections:

- *13. Assessing the sensitivity of new metrics p1 and p2 to cluster sizes*
- *14. Assessing the sensitivity of new metric p3 to the number of transition events*

I compliment the authors for making all the analysis files accessible through their github repository. However, it seems that none of the entire-lineage analyses converged (e.g. Delta_Thorney.log), which is worrisome. This makes me doubt the reliability of the resulting phylogenies, which are the basis for all of the following analyses?

Answer: We really thank the Reviewer for having detected this issue with the analyses shared in the GitHub repo of our study. As already outlined above in our answer to the Editor’s assessment, it appeared that the discrete and continuous phylogeographic analyses were indeed not based on Thorney BEAST analyses with proper convergence. We apologise for this issue that might have impacted some of the phylogeographic results we previously shared and, in particular for the Delta variant, for which the Thorney BEAST analysis had not even passed the burn-in. We tried to re-run longer Thorney BEAST analyses to reach sufficient MCMC convergence and mixing for all four variants, but this was not successful. Therefore, we opted to build on our previously published analytical pipeline (Dellicour *et al*. 2021, PMID: 33316043) and use the method implemented in the program TreeTime (Sagulenko *et al*. 2018, PMID: 29340210) for the time-calibration step. As detailed in our answer above to the Editor and second Reviewer (see above), this change in the analytical procedure presents another advantage: it now allows us to rely on previous work to discuss the limitation of basing the phylogeographic reconstruction on a unique time-scaled phylogeny.

Reviewer #2: The only major issue here is that the authors should consider accounting for inherent phylogenetic uncertainty by inferring even a modest distribution of trees (as other authors have done for phylogeographic studies of SARS-CoV-2) and then running their downstream phylogeographic analyses on this set of trees rather than relying upon a single topology.

Answer: We here refer to our answers to related comments above.

Part III – Minor Issues: Editorial and Data Presentation Modifications

Please use this section for editorial suggestions as well as relatively minor modifications of existing data that would enhance clarity.

Reviewer #1: Fig2: Why 80% HPD instead of the more established 95%?

Answer: Whereas it remains by definition an arbitrary choice, we estimated 80% HPD regions as it corresponds to a frequently used parameterization (e.g. Sahakyan *et al*. 2021, PMID: 33758277, and Viana *et al*. 2022, PMID: 35042229, for two recent examples) as well as the default visualisation option in tools (SPREAD and spreaD3, Bielejec *et al*. 2011, PMID: 21911333, 2016, PMID: 27189542) developed to visualise continuous phylogeographic reconstructions.

ll.160f "This probability": which probability is meant here?

Answer: “probability p1”; we have now clarified that sentence.

Reviewer #2: Line 25: “While the main research focus centred on the ability...” reword to be clear the authors mean the main focus of research more generally as worded it could be related to this effort, or the authors own main focus. “While the main focus of research has centred on the ability…”

Answer: Modification has been done.

Line 232: Change “This index has tended to be decrease over the epidemic waves…” to “This index has tended to decrease over the epidemic waves…”

Answer: Correction has been done.
